# Adaptive Modulation Tracking for High-Precision Time-Delay Estimation in Multipath HF Channels

**DOI:** 10.3390/s25144246

**Published:** 2025-07-08

**Authors:** Qiwei Ji, Huabing Wu

**Affiliations:** 1 National Time Service Center, Chinese Academy of Sciences, Xi’an 710600, China; jiqiwei21@mails.ucas.ac.cn; 2 University of Chinese Academy of Sciences, Beijing 100049, China; 3 Key Laboratory of Time Reference and Applications, Chinese Academy of Sciences, Xi’an 710600, China

**Keywords:** high frequency (HF), time-delay estimation (TDE), time-of-arrival (TOA), delay lock loop (DLL)

## Abstract

High-frequency (HF) communication is critical for applications such as over-the-horizon positioning and ionospheric detection. However, precise time-delay estimation in complex HF channels faces significant challenges from multipath fading, Doppler shifts, and noise. This paper proposes a Modulation Signal-based Adaptive Time-Delay Estimation (MATE) algorithm, which effectively decouples carrier and modulation signals and integrates phase-locked loop (PLL) and delay-locked loop (DLL) techniques. By leveraging the autocorrelation properties of 8PSK (Eight-Phase Shift Keying) signals, MATE compensates for carrier frequency deviations and mitigates multipath interference. Simulation results based on the Watterson channel model demonstrate that MATE achieves an average time-delay estimation error of approximately 0.01 ms with a standard deviation of approximately 0.01 ms, representing a 94.12% reduction in mean error and a 96.43% reduction in standard deviation compared to the traditional Generalized Cross-Correlation (GCC) method. Validation with actual measurement data further confirms the robustness of MATE against channel variations. MATE offers a high-precision, low-complexity solution for HF time-delay estimation, significantly benefiting applications in HF communication systems. This advancement is particularly valuable for enhancing the accuracy and reliability of time-of-arrival (TOA) detection in HF-based sensor networks and remote sensing systems.

## 1. Introduction

High-frequency (HF) communication, also known as short-wave communication, offers distinctive advantages including strong survivability, low power consumption, and wide coverage. Consequently, it plays an irreplaceable role in critical applications such as over-the-horizon positioning, time synchronization, and ionospheric sounding. Time-of-arrival (TOA) estimation constitutes a fundamental initial step in signal processing required to achieve high-precision positioning, time synchronization, and ionospheric sounding.

Traditional TOA estimation relies on signal correlation methods, including the Generalized Cross-Correlation (GCC) algorithm [1]. GCC estimates the time delay by identifying the peak of a weighted cross-correlation function between transmitted and received signals. This approach provides highly accurate TOA estimates under specific conditions: stationary additive Gaussian white noise and the assumption that the time intervals between multipath components exceed the correlation time of the transmitted signal. Alternatively, in scenarios characterized by a single signal component, the GCC method without weighting is equivalent to the maximum likelihood (ML) estimator, capable of delivering highly accurate TOA estimates. However, HF skywave signals propagate via the ionosphere, whose nonlinear time-varying characteristics induce detrimental effects such as multipath fading and Doppler frequency shifts. These effects significantly degrade the accuracy of HF signal delay parameter estimation.

Algorithms designed for multipath delay estimation can be broadly categorized as follows: For scenarios with known source signals, the multipath delay estimation problem is decomposed into multiple one-dimensional optimization problems through dimensionality reduction techniques for parameter estimation, with representative examples including the Expectation–Maximization (EM) algorithm [2], rooted in Maximum Likelihood Estimation (MLE), and the Weighted Fourier Transform and Relaxation (WR) algorithm [3], based on the Least Squares Method (LSM). When the source signal is unknown, the problem is transformed into its frequency-domain equivalent, resembling a sinusoidal signal frequency estimation problem, and subsequently addressed using subspace parameter estimation algorithms. Prominent algorithms in this category include the Multiple Signal Classification (MUSIC) algorithm [4,5,6] and the Estimation of Signal Parameters via Rotational Invariance Techniques (ESPRIT) algorithm [7,8]. Leveraging the inherent sparsity of delay parameters induced by the multipath effect, sparse reconstruction algorithms are employed for delay estimation. Representative algorithms in this class include the Sparse Iterative Covariance-based Estimation (SPICE) algorithm [9] and the Orthogonal Matching Pursuit (OMP) algorithm [10]. Furthermore, algorithms grounded in fractional-order Fourier transform theory offer novel approaches for multipath delay estimation of Linear Frequency-Modulated (LFM) signals [11], while deep learning methods present an emerging research direction in this domain.

However, existing research predominantly focuses on analyzing individual interference factors such as multipath effects or frequency shifts, lacking comprehensive models for composite interference mechanisms in HF channels. Moreover, subspace-based methods (e.g., MUSIC/ESPRIT) incur prohibitive computational complexity due to their reliance on matrix operations—specifically $O(N^{3})$ covariance decomposition—severely limiting real-time processing capabilities. In contrast, MATE achieves superior computational efficiency by confining FFT/IFFT operations to initial parameter acquisition and executing only multiplication/addition operations during the tracking phase (O(N) complexity). Carrier synchronization errors and modulation scheme variations further challenge conventional approaches in high-frequency time-of-arrival (TOA) estimation scenarios.

Addressing the aforementioned challenges, this paper introduces a novel HF time-delay estimation algorithm: the Modulation signal-based Adaptive Time-delay Estimation (MATE) algorithm, which leverages adaptive phase tracking. Specifically, MATE achieves carrier frequency deviation compensation and mitigates multipath interference through the decoupling of carrier and modulation signals, as well as the integration of phase-locked loop (PLL) techniques within the time-delay estimation framework. Compared to existing approaches, MATE eliminates the reliance on pseudo-random sequences and is directly applicable to non-spreading modulation schemes, exemplified by 8PSK. It demonstrates notable resilience against frequency-shift perturbations, robust performance under multipath conditions, and superior algorithmic generalizability. The proposed MATE algorithm thus provides a promising solution for high-precision time-delay sensing in practical HF communication and sensing scenarios, contributing to the development of more accurate and robust sensor systems reliant on HF signal propagation.

## 2. HF Time-Delay Estimation Signal Model

### 2.1. HF Channel Model

**Figure 1:**
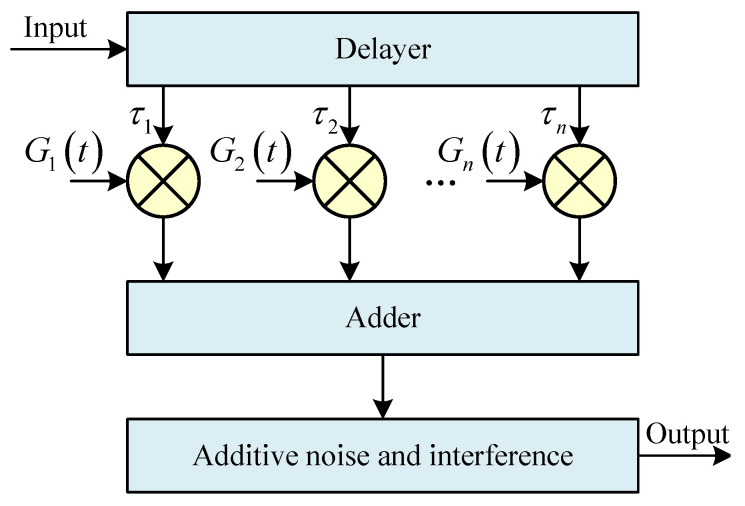
Block diagram of the Watterson model system.

The selection of the HF channel model significantly influences how closely the modeled channel approximates the actual channel characteristics. The Watterson model [12,13], a mathematical representation of the narrowband HF channel derived from empirical data, has been extensively utilized and is recommended by the ITU as a standard model. The parameters of this experimental simulation conform to the conditions of the Watterson model, thereby enabling the simulation of the HF channel using this model. The Watterson model is characterized as a Gaussian scattering tap gain delay line model, with its system block diagram illustrated in Figure 1:

In Figure 1, *i* denotes the ith propagation path, $\tau_{i}$ represents the time delay associated with the ith path, *n* is the total number of paths, and $G_{i}\left(t\right)$ signifies the stochastic gain process for the ith path. The input signal is first fed into a tap delayer, where each tap can be regarded as a propagation path through the ionosphere, and the multipath effect is simulated by varying the value of the time delay $\tau_{i}$. Then, different $G_{i}\left(t\right)$ modulate the amplitude and phase of the delayed signal to simulate Doppler shift and Doppler frequency spreading. Finally, the delayed and modulated individual signals are aggregated, with noise and interference added to produce the output signal [14].

The channel transfer function of the Watterson channel model is expressed as follows:

(1) $$h\left(f,t\right)=\sum_{i=1}^{T}G_{i}\left(t\right)e^{-j2\pi ft}$$

where *T* is the number of paths for multipath propagation; $G_{i}\left(t\right)$ is the channel gain function for the ith path, which is used to represent the effects of small-scale fading characteristics such as Doppler spreading, multipath fading, and Doppler frequency shift experienced by HF signals during transmission through the channel. $G_{i}\left(t\right)$ can be represented by a zero-mean complex Gaussian function:

(2) $$G_{i}\left(t\right)=G_{ia}\left(t\right)\exp\left(j2\pi f_{ia}t\right)+G_{ib}\left(t\right)\exp\left(j2\pi f_{ib}t\right)$$

where $G_{ia}\left(t\right)$ and $G_{ib}\left(t\right)$ are two mutually independent, complex Gaussian smooth stochastic processes with each state experienced; they are mutually independent orthogonal components with mean values of 0 and have equal root mean square values and spectra. The exponential factor is the Doppler shift of the two magneto-ionic components. The variance of $G_{ia}\left(t\right)$ and $G_{ib}\left(t\right)$ is the size of the Doppler expansion formed by the two magneto-ionic components, consisting of two mutually independent real Gaussian processes:

(3) $$G_{ia}\left(t\right)=g_{ia}\left(t\right)+j{\hat{g}}_{ia}\left(t\right)$$

$g_{ia}\left(t\right)$ and ${\hat{g}}_{ia}\left(t\right)$ have the same power spectral density.

The HF channel propagation patterns in practice are correlated and the statistical properties of the stochastic processes are time-varying, so the Watterson model is not applicable to bandwidths greater than 10 kHz and durations greater than 10 min.

### 2.2. Eight-Phase Shift Keying Signal Model

Within the domain of HF communication, due to the extremely tight spectrum resources, in order to effectively improve the utilization of the spectrum and data transmission efficiency, the 8PSK (Eight-Phase Shift Keying) modulation method has been widely used. The 8PSK method utilizes eight different phase states to characterize the 3-bit binary data, and its modulation signal can be expressed as follows:

(4) s(t)=Re[sbb(t)·ej2πfct]

where Re[·] denotes the operation of taking the real part, and $f_{c}$ is the carrier frequency. $s_{bb}\left(t\right)$ is the baseband signal, which can be expressed as follows:

(5) $$s_{bb}\left(t\right)=\sum_{k=0}^{M-1}d_{k}\cdot g\left(t-kT_{s}\right)$$

where $d_{k}\in \left\{e^{j2\pi m/8}|m=0,1,\cdots ,7\right\}$ are the 8PSK modulation symbols, which cover eight different phase states and are used to carry the information; g(t) is the shaping filter impulse response, which serves to shape the signal to meet the transmission requirements; and *M* is the number of codewords in a cycle.

**Figure 2:**
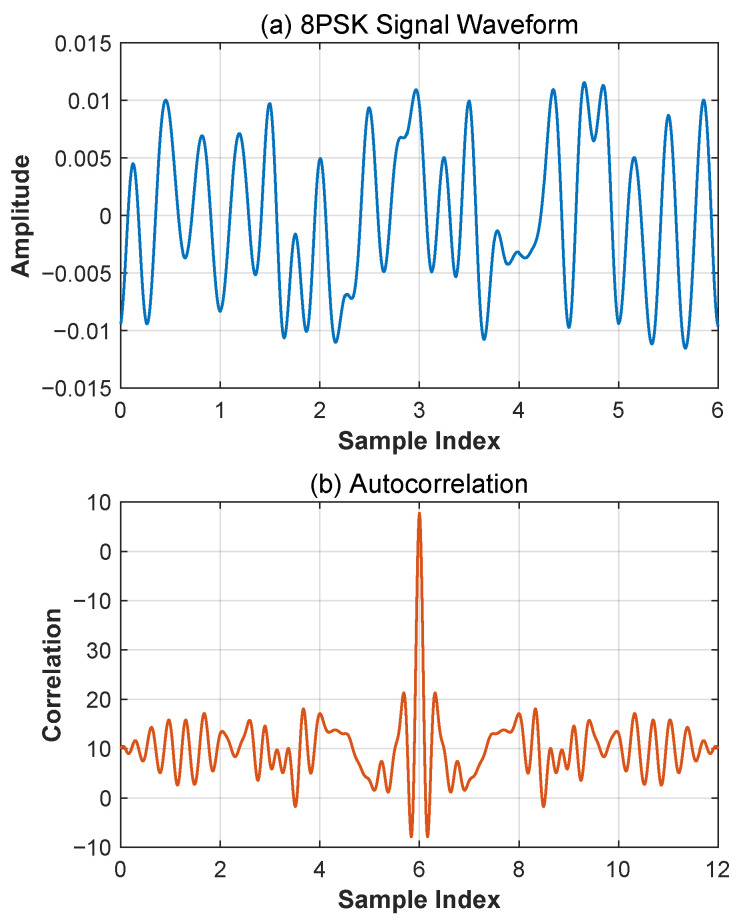
(**a**) Diagram of 8PSK modulated signal timing; (**b**) diagram of 8PSK modulated signal autocorrelation.

The transmit signal in the experimental simulation operates at a carrier frequency of 12.12 MHz, with 48 randomly generated code elements as a cycle continuously transmitted using 8PSK modulation. The signal baud rate is 2400 bps with a sampling rate of 30 MHz. Figure 2a shows the time-domain waveform of the 8PSK modulated signal, while Figure 2b displays its autocorrelation characteristics. These results clearly demonstrate that the 8PSK modulated signal exhibits pronounced autocorrelation properties, which establish a robust foundation for subsequent signal processing operations such as time-delay estimation in HF channel environments.

Combined with the Watterson HF channel model, the received signal model is given:

(6) $$y\left(t\right)=\sum_{i=1}^{T}G_{i}\left(t\right)s\left(t-\tau_{i}\right)+n\left(t\right)$$

where *T* is the number of paths for multipath propagation, and $G_{i}\left(t\right)$ is the channel gain function for the ith path. n(t) is Gaussian white noise; when the Doppler shift and Doppler spread caused by the two magnetic ion components are approximately the same, the channel gain function only needs to be expressed in terms of one magnetic ion component. Then, the channel gain function $G_{i}\left(t\right)$ can be simplified and expressed as follows:

(7) $$G_{i}\left(t\right)=G_{i}a\left(t\right)\exp\left(j2\pi f_{i}t\right)$$

## 3. MATE Algorithm

The MATE algorithm first requires initialization of the parameters. Drawing on the basic principle of the signal capture algorithm of the navigation receiver, the carrier frequency difference between the received signal and the transmitted signal, as well as the phase difference of the modulated signal, is estimated. Next, phase-locked loop technology is used to achieve the accurate tracking of the carrier frequency of the received signal and the phase of the modulated signal [15,16]. After the carrier stripping operation is completed, the time-of-arrival (TOA) is accurately estimated by precisely measuring the phase difference between the received and transmitted signals.

### 3.1. Initialization of MATE Parameters

**Figure 3:**
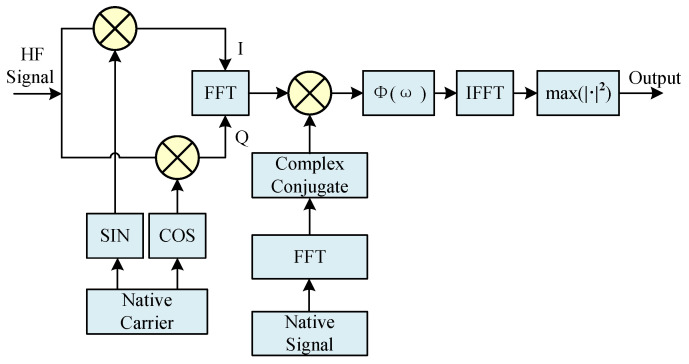
The structure of the MATE initialization algorithm.

The initialization method of MATE algorithm parameters refers to the signal capture algorithm of the navigation receiver and is combined with the generalized mutual correlation time-delay estimation method to increase the useful components of the signal by weighting the mutual correlation function, and the structure diagram of it is shown in Figure 3:

Φ(ω) in the figure denotes the weighting function, and here the Hassab–Boucher (HB) weighting function is used, which has better noise immunity [17], with the following expression:

(8) $$\Phi \left(\omega \right)=\frac{\left|G_{xy}\left(\omega \right)\right|}{G_{xx}\left(\omega \right)G_{xy}\left(\omega \right)}$$

where $G_{xy}\left(\omega \right)$ is the input signal and local signal cross-correlation function, and $G_{xx}\left(\omega \right)$ is the input signal autocorrelation function.

The algorithm systematically scans predefined frequency search intervals according to established frequency steps. Through exploiting the intrinsic relationship between Fast Fourier Transform (FFT) operations and circular convolution, it computes correlation values for each frequency bin. Subsequent analysis of these correlation metrics enables identification of the maximum autocorrelation peak, which facilitates the initial estimation of both carrier frequency offset and modulation phase discrepancy between transmitted and received signals [18].

### 3.2. TOA Tracking

**Figure 4:**
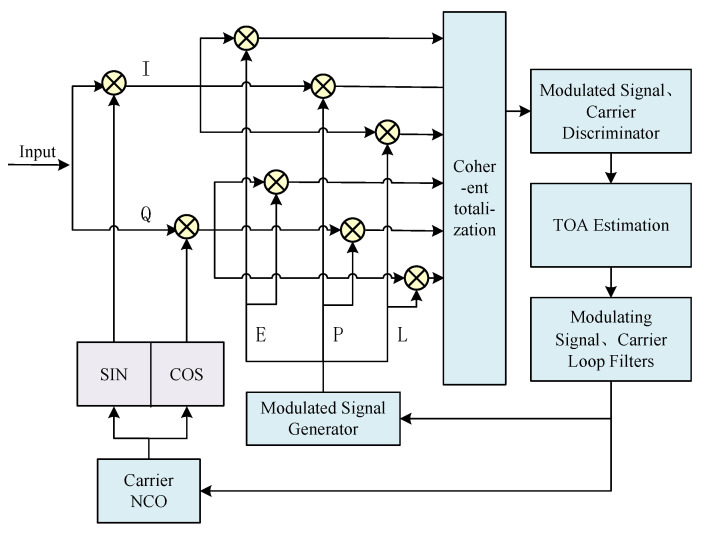
The structure of the TOA tracking.

After completing parameter initialization, the TOA tracking stage begins. The tracking loop employs closed-loop feedback mechanisms to achieve signal synchronization, and a good tracking loop design can accurately strip the carrier of the signal to obtain the phase deviation of the modulating signal [19], so as to realize high-precision delay estimation, so the tracking loop is the key link in realizing HF TOA. The structure diagram of the MATE tracking algorithm is shown in Figure 4.

TOA tracking consists of two parts: the modulating signal phase tracking loop and the carrier tracking loop. Among them, the carrier tracking loop can be divided into the phase-locked loop (PLL) and frequency-locked loop (FLL) [20], and the purpose of carrier tracking is to try its best to make the carrier signal it replicates consistent with the received HF carrier signal, so as to completely strip the carrier in the HF signal through frequency mixing [21]. The modulating signal phase tracking loop is mainly a delay-locked loop (DLL) [22], the main purpose of which is to calculate the phase difference between the received signal and the transmitted signal to accomplish TOA estimation. The main components of both loops are as follows: correlator, discriminator, loop filter, and local Numerically Controlled Oscillator (NCO) module.

The fundamental principle is outlined as follows: The received signal is first subjected to a mixing correlation operation with the in-phase (*I*) and quadrature (*Q*) carrier reference signals generated by the local carrier NCO. This critical step achieves both carrier stripping and signal down-conversion. Subsequently, the processed baseband signal is directed to three parallel correlation channels, where it undergoes precise correlation operations with the advanced (*E*), prompt (*P*), and delayed (*L*) modulation reference signals generated by the modulated signal generator. Through a meticulously designed integrate-and-dump operation (accumulating signal energy over a fixed time interval *T* followed by reset), six key correlation values are ultimately obtained: $I_{E}$, $Q_{E}$, $I_{P}$, $Q_{P}$, $I_{L}$, and $Q_{L}$. These correlation values can be uniformly expressed by the following mathematical expression:

(9) $$I=A\mathrm{sinc}\left(\pi \delta fT\right)*R\left(\delta \tau +kd\right)*\cos\left(\delta \tau \right)+n_{I}$$

(10) $$Q=A\mathrm{sinc}\left(\pi \delta fT\right)*R\left(\delta \tau +kd\right)*\sin\left(\delta \tau \right)+n_{Q}$$

where *A* is the signal amplitude; sinc(πδfT)=sin(πδfT)/(πδfT); δf is the carrier frequency error; *T* is the coherent integration time; R(·) is the modulating signal autocorrelation function; δτ is the modulating signal phase error; *d* is the neighbor correlator spacing; for the early, prompt, and late branches, k takes the values of −1, 0, and +1, respectively; and $n_{I}$ and $n_{Q}$ are the zero-mean Gaussian white noises of the two independently uncorrelated branches of I/Q, respectively.

Subsequently, the PLL, FLL, and DLL loops utilize the carrier phase discriminator, carrier frequency discriminator, and modulating signal phase discriminator to discriminate the tracking error of these six I/Q messages to obtain the carrier phase error, carrier frequency error, and modulating signal phase error, respectively. The algorithms used for each discriminator and their characterization are given in Table 1.

**Figure 5:**
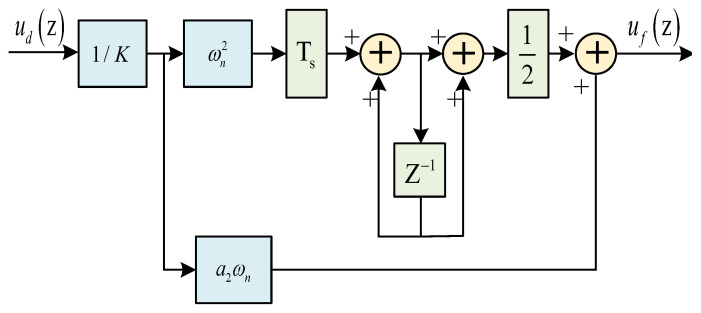
Block diagram of the loop filter.

The discriminator output discrimination error will be sent to the loop filter for low-pass filtering process to reduce the noise of the discrimination error in the loop. This helps avoid the high-frequency noise caused by over-regulation of the voltage-controlled oscillator and is conducive to the stability of the loop [25,26,27]. Figure 5 shows a block diagram of the second-order digital loop filter, and its filtering transfer function is as follows:

(11) $$F\left(z\right)=\frac{1}{K}\left(a_{2}\omega_{n}+\frac{T_{s}}{2}\frac{1+z^{-1}}{1-z^{-1}}\omega_{n}^{2}\right)$$

(12) $$a_{2}=2\xi$$

where $T_{s}$ is the data rate of the input filter instead of the sampling rate of the signal, *K* is the loop gain, $\omega_{n}$ is the eigenfrequency, and ξ is the damping factor.

The performance of the filter is affected by the loop bandwidth $B_{L}$ and the eigenfrequency $\omega_{n}$. The higher the $\omega_{n}$, the shorter the time it takes for the loop to reach the steady state. The narrower the $B_{L}$, the fewer frequency components are allowed to enter the loop, the better the filtering, and the more accurately the loop tracks the signal. However, $B_{L}$ should not be too small; too small a $B_{L}$ may filter out high-frequency components that vary normally in frequency and phase. Typical second-order loop filter parameters are as follows:

(13) $$a_{2}=1.414$$

(14) $$B_{L}=\frac{1+a_{2}^{2}}{4a_{2}}\omega_{n}=0.53\omega_{n}$$

## 4. Simulation Results and Experimental Verification

### 4.1. Simulation Results

In order to verify the performance of the MATE algorithm in a complex HF channel environment, this paper designs simulation experiments based on the Watterson channel model. The transmitted signal is a set of randomly generated 48 integers ([0, 8)), modulated by 8PSK, with a center frequency of 12.12 MHz and a bandwidth of 2.4 kHz, and the received signal sampling rate is 30 MHz.

The multipath effect and Doppler shift were simulated in the experiments, with the number of paths set to 2 and a delay difference of 10 ms. To evaluate the robustness of the algorithms, 140 sets of HF data were randomly generated, with the signal-to-noise ratio set to 0 dB, and the maximum Doppler shift varied randomly between 0 Hz and 20 Hz. The delay of the first path was set to 0, and the power was randomly distributed between 0 dB and −4 dB; the delay of the second path varied randomly between 0.0001 s and 0.002 s, and the power was randomly distributed between −5 dB and −9 dB.

**Figure 6:**
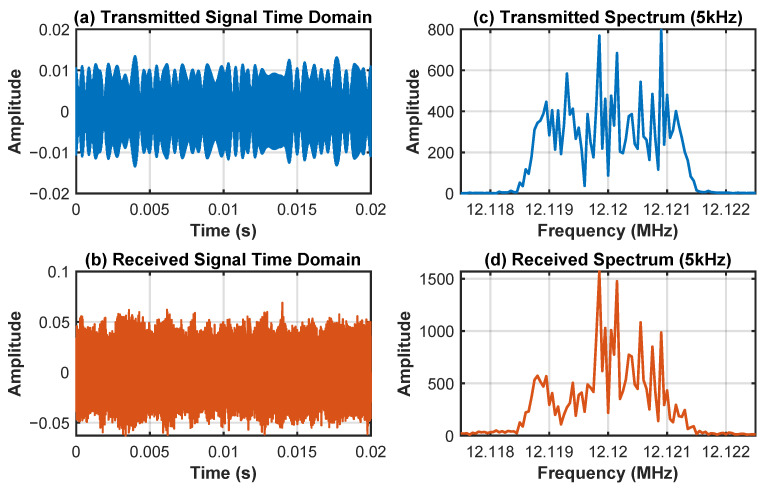
Signal comparison; (**a**) transmitted signal time domain; (**b**) received signal time domain; (**c**) frequency domain of the transmitted signal; (**d**) frequency domain of the received signal.

Figure 6 illustrates the time- and frequency-domain comparison of the first set of transmitted and received signals. The transmitted signals (Figure 6a,c) have clear 8PSK modulation characteristics, while the received signals (Figure 6b,d) exhibit obvious multipath fading and frequency-shift characteristics due to the processing of the Watterson channel model, verifying the complex interference effect of the HF channel on the signals.

**Figure 7:**
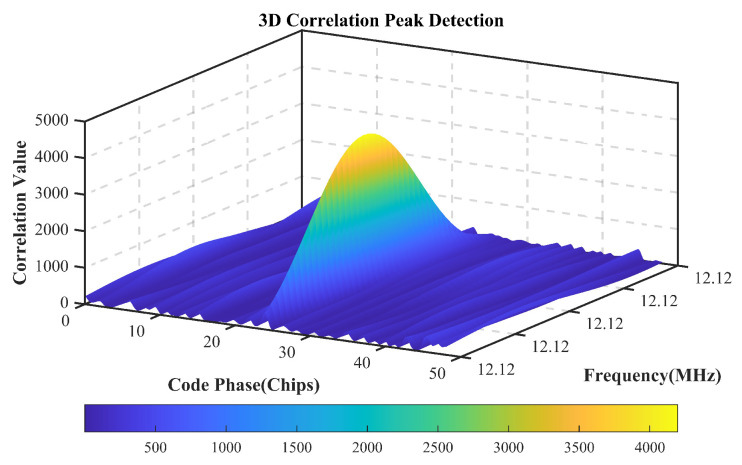
Acquisition results.

**Figure 8:**
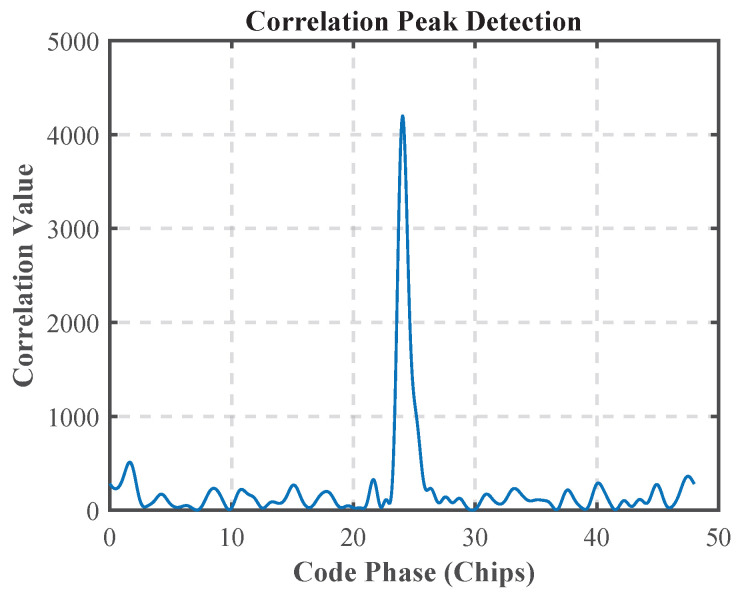
Corresponding values for acquisition frequency.

Figure 7 and Figure 8 demonstrate the initialization results of the MATE algorithm. The acquisition results show that the method is able to initially estimate the carrier frequency of the multipath signal and the phase of the modulating signal to achieve the initialization of the parameters of the MATE algorithm.

**Figure 9:**
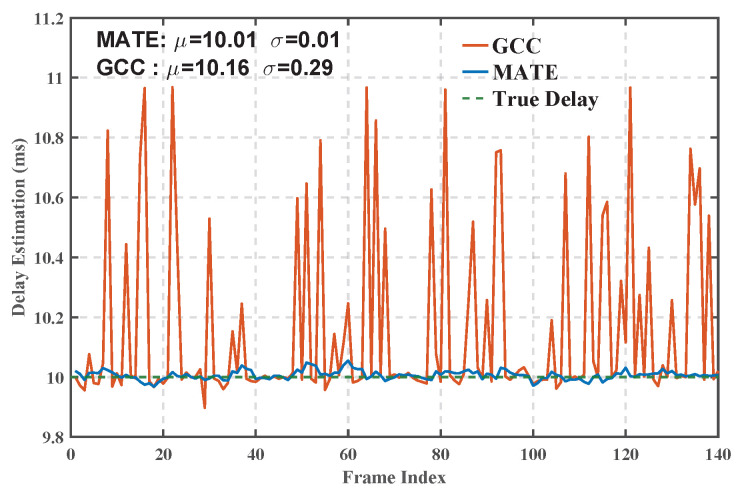
Time-delay estimation results.

**Figure 10:**
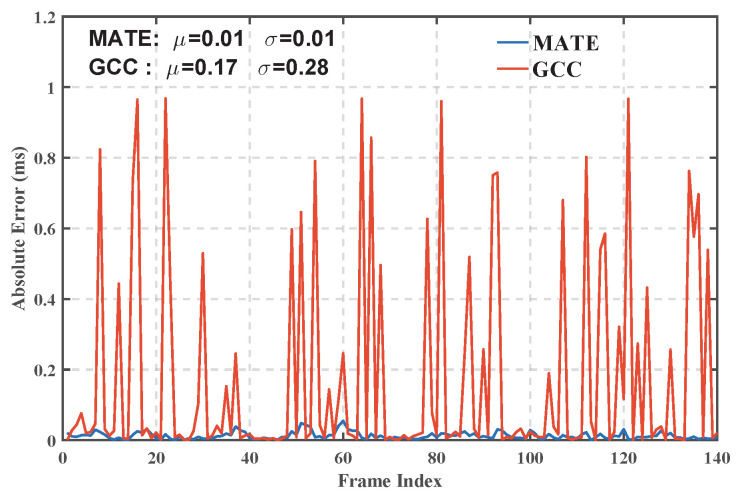
Time-delay estimation error results.

Figure 9 and Figure 10 show the delay estimation results and their error analysis of the MATE algorithm and the traditional generalized cross-correlation algorithm (GCC), respectively. The experimental results show that the average delay estimation error of the MATE algorithm represents a 94.12% reduction in mean error and a 96.43% reduction in standard deviation compared to the traditional Generalized Cross-Correlation (GCC) method. This result fully proves the superiority of the MATE algorithm in high-precision delay estimation in the complex HF channel environment and provides a more accurate and effective solution to the delay estimation problem in the field of HF communication.

### 4.2. Verification of Actual Measurement Data

The measured data are collected from the HF communication link from Xi’an, Shaanxi Province to Urumqi, Xinjiang Province, and the transmitted signal is modulated by 8PSK with 196 code elements as one transmission cycle, the data length is 86 cycles, the center frequency is 12.36 MHz, the signal bandwidth is 9.6 kHz, and the sampling rate at the receiving is set to 120 MHz. The measured scenario covers the typical ionospheric propagation characteristics, and it has the characteristics of multipath effects and a slowly time-varying channel.

In order to verify the actual performance of the MATE algorithm, it is analyzed in comparison with the GCC algorithm. The correctness of the algorithm is verified by analyzing the outputs of different branches, considering the real delay agnostic to the real test environment.

**Figure 11:**
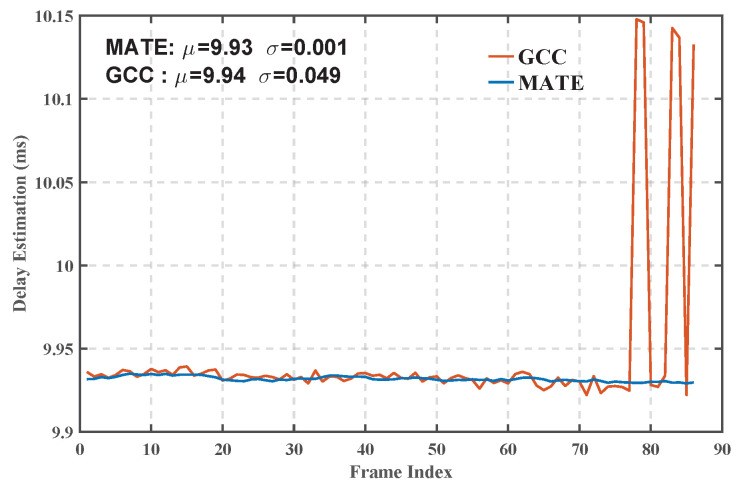
Time-delay estimation results.

Figure 11 shows the comparison of the delay estimation results of the two algorithms. The data show that the fluctuation amplitude of the delay estimation curve of the MATE algorithm (σ = 0.001 ms) is significantly lower than that of the GCC algorithm (σ = 0.049 ms), which reflects a stronger resistance to channel time-varying capability. In particular, the GCC algorithm shows significant estimation bias after the 75th cycle, which is due to the attenuation of the first arriving path gain as a result of the dynamic changes in the HF channel, whereas the GCC relies on the single-path correlation peak detection, which is susceptible to the change in the intensity of the multipath components.

**Figure 12:**
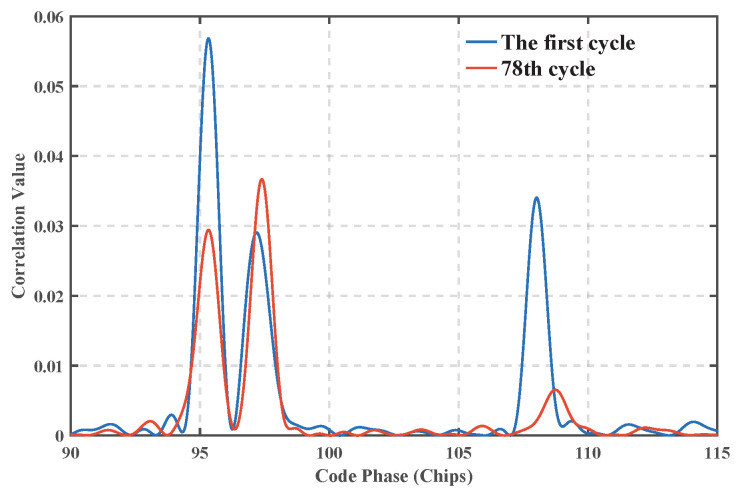
Corresponding values for acquisition frequency.

Figure 12 compares the distribution of correlation peaks corresponding to the acquisition frequency of the HF data in the 1st and 78th cycles, and it can be seen that the correlation peak energy of the first reach path in the 78th cycle decreases by about 50% compared with that in the 1st cycle, confirming the degradation of the first reach path gain due to channel fading, which is directly correlated to the fluctuation in the estimation of the GCC algorithm in this time period.

**Figure 13:**
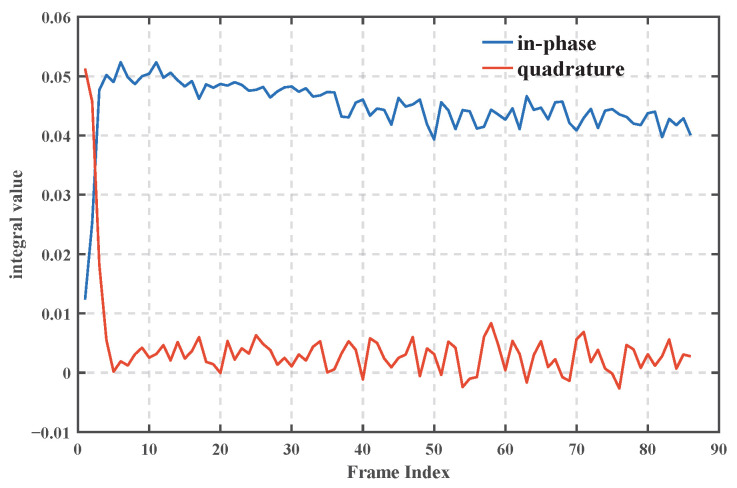
Comparison of in-phase and quadrature energy.

**Figure 14:**
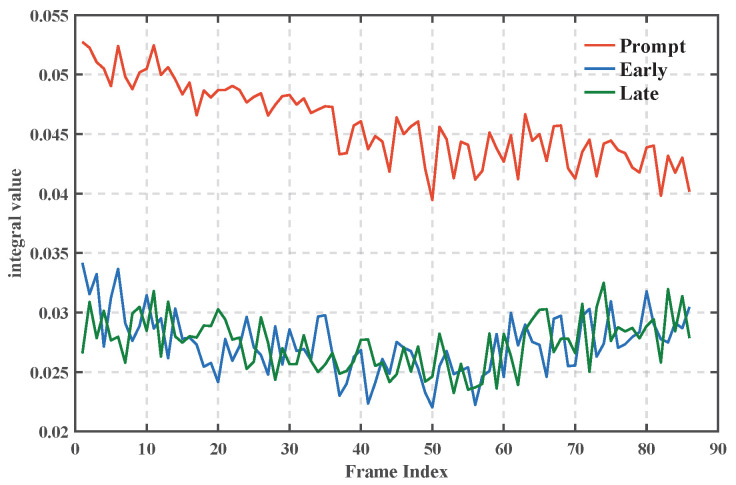
Comparison of Prompt–Early/Late Energy.

Studies [28,29] have shown that when the algorithm successfully locks the signal, the Prompt branch energy should be about twice as much as the Early and Late branches, and the in-phase component (I-path) is dominant, while the quadrature component (Q-path) tends to be close to zero. Figure 13 shows that the in-phase integral value of the Prompt branch of the MATE algorithm is stable at about 0.05, and the quadrature value is close to 0. In Figure 14, the energy of the Prompt branch is about twice that of the Early/Late branch, which verifies the effectiveness of the tracking loop and the accuracy of the phase estimation.

**Figure 15:**
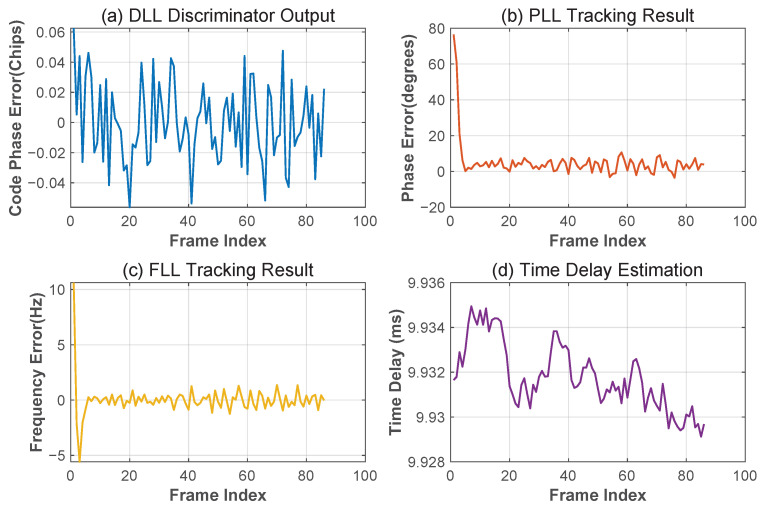
Tracking results. (**a**) Modulated signal phase tracking results; (**b**) carrier phase tracking results; (**c**) carrier frequency tracking results; (**d**) delay estimation results.

Figure 15 shows the key parameters of the tracking loop: the modulated signal number phase error (DLL output) is stable within ±0.06 code slice, and the carrier phase error (PLL output) and carrier frequency error (FLL output) are close to 0, which indicates that the loop successfully achieves carrier stripping and phase locking. The MATE delay estimation values in Figure 15d show a narrow band distribution around the mean value of 9.93 ms, which further verifies the high-accuracy characteristics of the algorithm in real channels.

### 4.3. Analysis and Comparison of Algorithm Complexity

The MATE algorithm employs a stage-wise processing strategy. The initialization phase (i.e., the first signal cycle) achieves parameter acquisition through FFT and IFFT operations, while subsequent cycles enter the tracking phase requiring only addition and multiplication operations. In contrast, the GCC algorithm necessitates FFT and IFFT computations every cycle.

Table 2 presents a comparative analysis of the time complexity and execution time for both algorithms, measured on the MATLAB R2022a platform using an AMD Ryzen 7 7735H processor with Radeon Graphics@ 3.20 GHz. The processor is manufactured by Advanced Micro Devices, Inc. (AMD), whose headquarters are located in Santa Clara, CA, USA.

In Table 2, *K* represents the number of signal periods, and *N* represents the number of sampling points per signal period. Clearly, both the time complexity and running time of the MATE algorithm are significantly lower than those of the GCC algorithm. The measured execution time for processing the same dataset confirms this substantial efficiency improvement, with MATE requiring approximately 57.67% less time than the GCC approach.

## 5. Discussion

This study proposes the Modulation Signal-based Adaptive Time-delay Estimation (MATE) algorithm. MATE achieves significant improvements in time-delay estimation accuracy within complex HF channels by decoupling carrier and modulation signals, as well as integrating PLL and DLL techniques. Simulation results based on the Watterson channel model demonstrate that MATE achieves an average time-delay estimation error of approximately 0.01 ms with a standard deviation of approximately 0.01 ms. Compared to the traditional GCC method, MATE represents a 94.12% reduction in mean error and a 96.43% reduction in standard deviation.

This performance enhancement is attributed to MATE’s effective utilization of the autocorrelation properties of 8PSK signals, enabling compensation for carrier frequency offsets and suppression of multipath interference. Crucially, compared to existing approaches, MATE eliminates the dependency on pseudo-random sequences and is directly applicable to non-spreading modulation schemes such as 8PSK. This overcomes a key limitation of conventional methods, which often rely on specific signal formats, and demonstrates superior resilience against frequency-shift perturbations, robustness under multipath conditions, and enhanced algorithmic generalizability.

Validation using actual measurement data from the Xi’an–Urumqi HF link confirmed the algorithm’s robustness in real-world ionospheric environments. Notably, when the energy of multipath components varied dynamically, the GCC algorithm exhibited significant estimation bias due to its reliance on single-path correlation peak detection. In contrast, MATE’s tracking loop, incorporating real-time phase locking, maintained estimation stability, with its delay estimation fluctuation (σ=0.001 ms) being substantially lower than that of GCC (σ=0.049 ms).

However, this study has certain limitations. Primarily, the current algorithm is designed for narrowband HF channels (bandwidth ≤ 10 kHz), and its applicability to broadband scenarios requires further investigation. Furthermore, although MATE outperforms traditional methods in multipath environments, its resolution capability in dense multipath scenarios remains constrained by the signal’s autocorrelation properties, potentially leading to estimation ambiguities.

Future research will explore extending MATE’s applicability to higher-order modulation schemes such as QAM to enhance cross-scenario adaptability, investigate the integration of sparse reconstruction techniques (e.g., Orthogonal Matching Pursuit algorithm) to improve resolution in dense multipath environments, examine fusion with deep learning methodologies to leverage neural networks for robust feature learning and noise modeling, and address broadband challenges through extended HF channel models and corresponding algorithmic adaptations.

The findings of this study provide crucial technical support for HF applications such as over-the-horizon positioning and ionospheric sounding. MATE offers a high-precision, robust, and relatively low-complexity solution to the time-delay estimation problem in complex HF channels. More importantly, the enhanced precision and robustness of MATE in estimating signal time-of-arrival directly translate to improved accuracy and reliability for a wide range of sensor systems that rely on HF signal propagation for timing, positioning, or environmental sensing. This includes distributed HF sensor networks for remote monitoring and HF-based remote sensing platforms, where precise TOA detection is fundamental to data integrity and system performance.

## Figures and Tables

**Table 1:** Discriminator algorithms and characteristics.

Type	Discriminator Algorithm	Characterization
carrier wave phase discriminator	$\arctan(Q_{P}/I_{P})$	The output is independent of the signal amplitude and is computationally intensive but accurate [23].
carrier wave frequency discriminator	$\varphi_{e}\left(n\right)-\varphi_{e}\left(n-1\right)/T$, where $\varphi_{e}\left(n\right)$ is the phase difference angle, $\varphi_{e}\left(n-1\right)$ is the phase difference angle of the n-1 th calendar element, and *T* is the coherent integration time.	The output result is independent of the signal amplitude, and the frequency discrimination is accurate but the calculation is large [24].
modulated signal phase discriminator	(1−d)∗(E−L)/(E+L) where *d* is the correlator spacing. $E=\sqrt{I_{E}^{2}+Q_{E}^{2}}$, $L=\sqrt{I_{L}^{2}+Q_{L}^{2}}$	The normalized incoherent presubtracted back-envelope method with a linear region of operation is [−d, d] [22].

**Table 2:** Comparison of algorithm complexity.

Algorithm	Computational Complexity	Running Time of Measured Data (s)
MATE	O(NlogN)+(K−1)O(N)	80.22
GCC	KO(NlogN)	189.52

## Data Availability

The supporting data for this study can be obtained upon request from the corresponding author. Due to privacy concerns involving the participants, these data are not publicly available.
